# Supplementary material for: The interactome of intact mitochondria by cross-linking mass spectrometry provides evidence for coexisting respiratory supercomplexes
Source: Mol Cell Proteomics. 2017 Dec 8;17(2):216–32. doi: 10.1074/mcp.RA117.000470 (PMC5795388; doi:10.1074/mcp.RA117.000470)
Supplement: Supplemental Data [file supp_RA117.000470_133922_0_supp_23978_fzffwf.zip › spectra_annotation/mito_spectra_annotation/202-1-1-1-2-1.pdf]

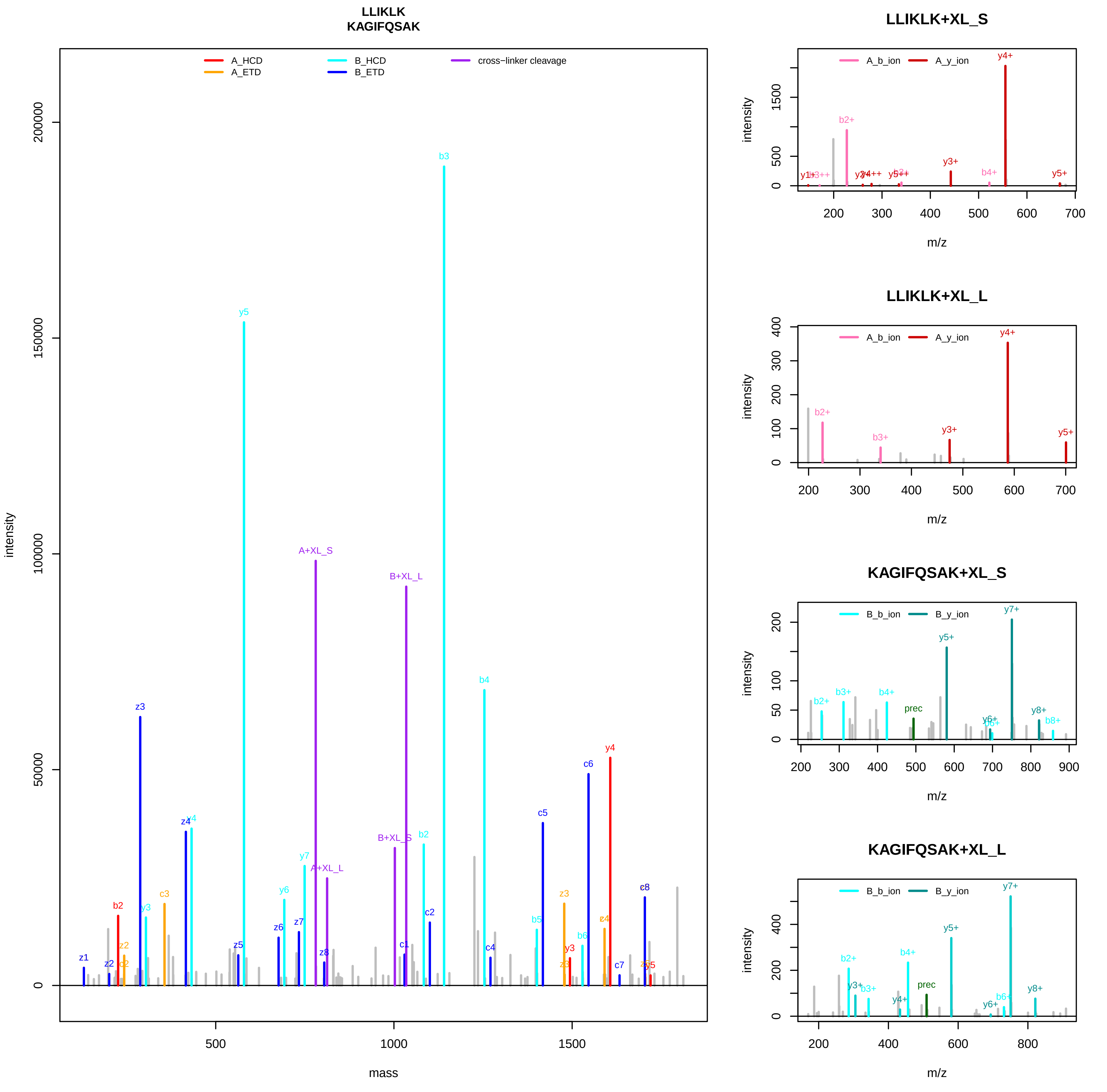

LLIKLK
KAGIFQSAK
A_HCD
A_ETD
B_HCD
B_ETD
cross−linker cleavage
intensity
mass
0
50000
100000
150000
200000
500
1000
1500
b3
y5
A+XL_S
B+XL_L
b4
z3
y4
c6
c5
z4
y4
B+XL_S
b2
y7
A+XL_L
y6
c8
b2
y3
c3
z3
c2
z4
b5
z6
z7
b6
z5
z2
c2
z8
c1
c4
y3
z1
z2
c7
y5
LLIKLK+XL_S
A_b_ion
A_y_ion
y4+
b2+
y3+
b4+
y5+
intensity
m/z
0
500
1500
200
300
400
500
600
700
LLIKLK+XL_L
A_b_ion
A_y_ion
y4+
b2+
b3+
y3+
y5+
intensity
m/z
0
100
200
300
400
200
300
400
500
600
700
KAGIFQSAK+XL_S
B_b_ion
B_y_ion
y7+
y5+
b2+
b3+
b4+
prec
y6+
y8+
b8+
intensity
m/z
0
50
100
200
200
300
400
500
600
700
800
900
KAGIFQSAK+XL_L
B_b_ion
B_y_ion
y7+
y5+
b2+
b4+
prec
y3+
b3+
y4+
y6+
b6+
y8+
intensity
m/z
0
200
400
200
400
600
800
